# Comparative study of ultrasound attenuation analysis and controlled attenuation parameter in the diagnosis and grading of liver steatosis in non-alcoholic fatty liver disease patients

**DOI:** 10.1186/s12876-024-03160-8

**Published:** 2024-02-23

**Authors:** Mengyun Wang, Shuzhen Tang, Guoqiu Li, Zhibin Huang, Sijie Mo, Keen Yang, Jing Chen, Baishan Du, Jinfeng Xu, Zhimin Ding, Fajin Dong

**Affiliations:** 1 The Second Clinical Medical College, Jinan University, Guangzhou, China; 2 Department of Ultrasound, The Second Clinical Medical College, Jinan University (Shenzhen People’s Hospital), Shenzhen, China

**Keywords:** Ultrasound attenuation analysis, Hepatic steatosis, Controlled attenuation parameters, Non-alcoholic fatty liver disease

## Abstract

**Purpose:**

To assess the diagnostic performance of Ultrasound Attenuation Analysis (USAT) in the diagnosis and grading of hepatic steatosis in patients with non-alcoholic fatty liver disease (NAFLD) using Controlled Attenuation Parameters (CAP) as a reference.

**Materials and methods:**

From February 13, 2023, to September 26, 2023, participants underwent CAP and USAT examinations on the same day. We used manufacturer-recommended CAP thresholds to categorize the stages of hepatic steatosis: stage 1 (mild) − 240 dB/m, stage 2 (moderate) − 265 dB/m, stage 3 (severe) − 295 dB/m. Receiver Operating Characteristic curves were employed to evaluate the diagnostic accuracy of USAT and determine the thresholds for different levels of hepatic steatosis.

**Results:**

Using CAP as the reference, we observed that the average USAT value increased with the severity of hepatic steatosis, and the differences in USAT values among the different hepatic steatosis groups were statistically significant (*p* < 0.05). There was a strong positive correlation between USAT and CAP (*r* = 0.674, *p* < 0.0001). When using CAP as the reference, the optimal cut-off values for diagnosing and predicting different levels of hepatic steatosis with USAT were as follows: the cut-off value for excluding the presence of hepatic steatosis was 0.54 dB/cm/MHz (AUC 0.96); for mild hepatic steatosis, it was 0.59 dB/cm/MHz (AUC 0.86); for moderate hepatic steatosis, it was 0.73 dB/cm/MHz (AUC 0.81); and for severe hepatic steatosis, it was 0.87 dB/cm/MHz (AUC 0.87).

**Conclusion:**

USAT exhibits strong diagnostic performance for hepatic steatosis and shows a high correlation with CAP values.

## Introduction

Non-alcoholic fatty liver disease (NAFLD) is the most common chronic liver disease worldwide. It has been reported that the global prevalence of NAFLD is as high as 25.24%, and in some regions, it has exceeded 50% [1]. In 2018, the prevalence in China was approximately 29.2% [2], making it the most common chronic liver disease in China, surpassing viral hepatitis [3]. NAFLD is a disease characterized by excessive accumulation of triglycerides in the liver, leading to hepatic steatosis. It is a dynamic process that progresses from simple fat accumulation to non-alcoholic steatohepatitis (NASH) and may ultimately result in serious consequences such as cirrhosis and liver cancer [4]. Moreover, NAFLD is closely associated with factors like obesity and diabetes, increasing the risk of cardiovascular diseases, kidney diseases, and certain cancers [5, 6]. Therefore, accurate diagnosis and quantitative analysis of hepatic steatosis are essential and can effectively reverse hepatic steatosis, hepatitis, and fibrosis.

Hepatic steatosis is a prerequisite for diagnosing NAFLD, and liver biopsy is considered the “gold standard” for diagnosing and grading hepatic steatosis. However, biopsy is an invasive procedure that can lead to complications [7], and there is a risk of sample errors as it only samples a small portion of the liver and may not reflect the overall liver condition, potentially leading to misdiagnosis [8]. Considering these limitations, non-invasive diagnostic methods have emerged, including ultrasound, computed tomography (CT), and magnetic resonance imaging (MRI). Conventional ultrasound is the most common liver imaging method used to identify hepatic steatosis, relying on multiple factors such as liver echogenicity, the degree of attenuation of posterior echoes, portal vein wall echo, and diaphragmatic muscle clarity. However, conventional ultrasound diagnosis is subjective and has limited sensitivity for mild hepatic steatosis. Moreover, its assessment of hepatic steatosis can be influenced by severe fibrosis [9, 10]. CT also has limited sensitivity for quantifying hepatic steatosis, with high diagnostic accuracy achieved only when hepatic fat infiltration is greater than 30%, and it carries a risk of radiation exposure [11]. Although MRI can accurately display the degree of hepatic fat deposition, it is relatively costly, requires a longer examination time, and may be impractical in patients with obesity [12].

In recent years, FibroScan has developed Controlled Attenuation Parameter (CAP), a technology based on the characteristics of ultrasound signals. CAP quantitatively evaluates hepatic steatosis by measuring liver stiffness and liver attenuation parameters [13]. Research results have shown that CAP has excellent diagnostic value for NAFLD patients and can detect hepatic fat infiltration greater than 5% [14]. Additionally, CAP has the advantages of being non-invasive, easy to perform, providing immediate results, and being cost-effective. Asia-Pacific guidelines recommend CAP as a screening tool for NAFLD [15, 16]. However, different studies have reported variations in the diagnostic threshold and efficiency of CAP for assessing the degree of steatosis. Furthermore, CAP lacks ultrasound image’s guidance, making it unable to dynamically observe the liver parenchyma in the examination area, and measurements may be influenced by the biliary system, large blood vessels, or liver lesions [17, 18]. Therefore, there is an urgent need in clinical practice for a quantitative examination technology that is accurate, efficient, and integrates liver visual structure with two-dimensional acoustic attenuation results.

Recently, a new technology called Ultrasound Attenuation Imaging (USAT), which uses attenuation coefficients to quantitatively detect hepatic steatosis. USAT is based on the “whole-domain sound field restoration” technology of the original ultrasound radiofrequency signal, which restores the true sound speed and original attenuation information at various positions in the liver, effectively improving the accuracy and repeatability of measurements.

Therefore, USAT can be used for screening, quantitative diagnosis, and follow-up of NAFLD.

## Methods and materials

### Patient selection

Ethical approval was obtained from the Institutional Review Board of Shenzhen People’s Hospital. Written informed consent was obtained from all study participants. From February 13, 2023, to September 26, 2023, 212 patients were recruited at Shenzhen People’s Hospital to participate in our study. Inclusion criteria for NAFLD participants were: (1) diagnosed with or suspected of having NAFLD; (2) age ≥ 18 years; (3) all participants underwent USAT and CAP examinations on the same day; (4) collection of age, gender, body mass index, medical history, and alcohol consumption status. Exclusion criteria for NAFLD participants were: (1) heavy alcohol consumption (≥ 14 drinks per week for men, ≥ 7 drinks per week for women); (2) presence of liver diseases other than NAFLD; (3) the use of fat-preparation (including fat supplements, fat-based medications, high-fat foods, or beverages, and other potential fat interventions) or hepatotoxic drugs; (4) presence of significant systemic illnesses or any other conditions that the researchers believed would affect the patient’s ability, compliance, or completion of the study. (5) the presence of factors that may lead to CAP errors, such as cytolysis, cholestasis, congestion, amyloidosis, lymphomas, and extramedullary hematopoiesis. The inclusion and exclusion criteria for normal participants were the same as for NAFLD participants, except they had no history of NAFLD.

### Examiners and medical equipment

CAP and USAT examinations were conducted by an expert with 20 years of experience in liver ultrasound testing. The expert was unaware of the clinical diagnosis of the patients. USAT measurements were performed using the Resona 7 system (Mindray, probe SC6-1U, Shenzhen, China), and CAP measurements were conducted using the FibroScan Handy (Echosens, probes M and XL, Paris, France).

### Examination procedures

**Fig. 1:**
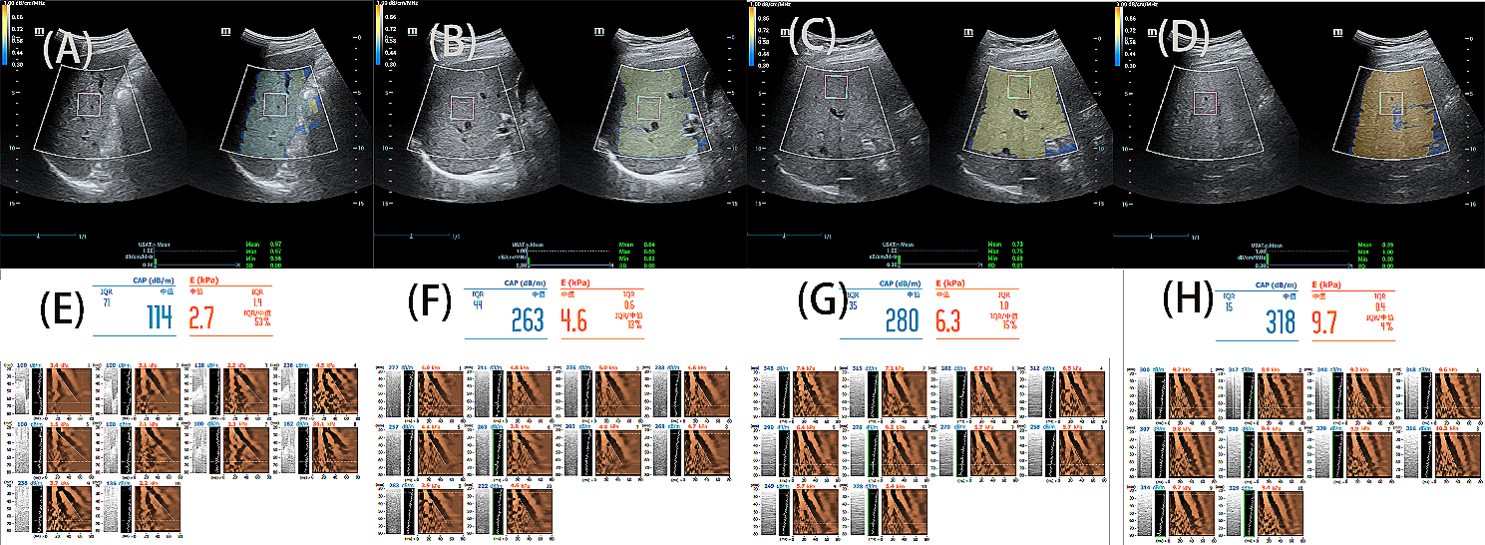
Samples of four subjects. From **A** to **D**, representing USAT measurements for no, mild, moderate, and severe hepatic steatosis; from **E** to **H**, representing CAP measurements for no, mild, moderate, and severe hepatic steatosis. CAP, controlled attenuation parameter; USAT,ultrasound attenuation analysis

All participants fasted for more than eight hours, and each subject completed the above examinations on the same day. During the scan, participants were placed in a supine position with their right upper limb resting on their head. When measuring CAP, the expert measured the intercostal spaces at the anterior axillary line or mid-axillary line, obtaining at least 10 valid individual measurements. Finally, the median of the valid measurement data was used as the representative CAP value, expressed in dB/m. When measuring USAT, the expert placed the probe in the right lobe of the subject’s liver, with the upper edge of the sampling frame located 1–2 cm below the liver capsule. USAT values were measured when the sampling frame was filled with yellow. The results were expressed in dB/cm/MHz. Detailed explanations of the USAT technique can be found in the Mindray white paper (Fig. 1).

### Hepatic steatosis grade definition

The grade classification of hepatic steatosis is based on CAP values: normal < 240 dB/m; mild steatosis 240 dB/m ≤ CAP < 265 dB/m; moderate steatosis 265 dB/m ≤ CAP < 295 dB/m; severe steatosis ≥ 295 dB/m. When there was a significant deviation between CAP and USAT values for some participants, the final diagnosis was determined by the expert with 20 years of experience in liver ultrasound scanning, deciding whether to adhere to their diagnosis or use the CAP value as a reference diagnosis.

### Statistical analysis

Statistical analysis was performed using R software 4.3.1 (https://www.r-project.org), and graphs were created using GraphPad Prism version 9.0.0 for Windows (GraphPad Software, San Diego, California USA). Continuous numerical variables were expressed as means ± standard deviations or medians and interquartile ranges (IQR) based on their distribution, and categorical variables were expressed as the number of patients and proportions. The normality of the distribution of continuous variables was tested using the Shapiro-Wilk test. The Kruskal-Wallis test was used to analyze the significance of differences between groups. The Spearman rank correlation coefficient was used to assess the correlation between USAT and CAP [19]. Receiver Operating Characteristic (ROC) curve analysis was used to evaluate diagnostic performance and thresholds. For each ROC analysis, the area under the curve (AUC), cut-off value, sensitivity, specificity, and accuracy were calculated. A *p*-value < 0.05 was considered statistically significant.

## Results

### Patient baseline information

A total of 212 participants were included in this study, comprising 75 females (35%) and 137 males (65%), with a mean age of 41 years. When using *P* < 0.05 as the criterion, there were statistically significant differences in ALT (*P* < 0.0001), AST (*P* = 0.0015), ALP (*P* = 0.0314), HDL (*P* = 0.0110), BMI (*P* < 0.0001), USAT (*P* < 0.0001), and CAP (*P* < 0.0001) between the normal control group and the hepatic steatosis group. No significant differences were observed in age, gender, PLT, ALB, TP, DB, GLB, and LDL variables between the two groups (Table 1).

**Table 1:** Patient baseline information

Variables	Total (*n* = 212)	Stage0 (*n* = 80)	Stage1 (*n* = 46)	Stage2 (*n* = 35)	Stage3 (*n* = 51)	*p*
Age, years	41.00 (32.75, 51.25)	41.00 (32.75, 51.25)	41.00 (32.25, 47.75)	45.00 (34.00, 52.00)	39.00 (32.50, 53.00)	0.7590
Sex, n (%)						0.2219
Female	75.00 (35.00)	35.00 (44.00)	15.00 (33.00)	9.00 (26.00)	16.00 (31.00)	
Male	137.00 (65.00)	45.00 (56.00)	31.00 (67.00)	26.00 (74.00)	35.00 (69.00)	
PLT,×10^9/L	243.00 (209.00, 282.00)	239.00 (211.00, 270.00)	236.50 (209.75, 294.75)	247.50(207.00, 258.00)	247.00 (199.00, 271.00)	0.9474
ALT, U/L	27.00 (18.00,51.25)	19.50 (14.45, 34.25)	25.00 (19.00, 35.00)	36.00 (22.00, 49.00)	49.50(28.00, 71.00)	< 0.0001
AST, U/L	24.00 (19.00, 33.00)	21.5(18.75, 29.25)	22.00 (19.00, 26.00)	26.00 (22.00,32.00)	31.50(22.75, 48.50)	0.0015
ALP, U/L	76.00 (63.00, 91.00)	73.00 (59.00, 89.00)	76.00 (68.00, 86.00)	74.00 (65.00, 89.00)	88.00 (70.00, 101.00)	0.0314
ALB, g/L	44.35 (42.08, 46.7)	43.75 (40.1, 46.32)	44.90 (43.50, 46.70)	44.80 (43.10, 47.20)	44.55 (43.38, 46.78)	0.1006
TP, g/L	74.70 (70.47, 78.03)	73.95 (67.35, 76.65)	73.90 (70.75, 77.75)	76.10 (72.5, 77.80)	76.15 (71.98, 79.22)	0.0731
DB, umol/L	2.50 (1.80, 3.10)	2.60 (1.80, 3.50)	2.55 (2.00, 3.10)	2.20 (1.78,2.70)	2.60 (1.90, 3.30)	0.4324
GLB, g/L	30.00 (26.00, 33.45)	30.50 (26.85, 34.00)	30.00 (26.00, 32.50)	30.00 (25.75, 32.00)	30.50 (27.75, 34.00)	0.4532
LDL, umol/L	2.74 (2.28, 3.27)	2.75 (2.36, 3.14)	2.74 (2.37, 3.32)	2.88 (2.52, 3.07)	2.66 (2.09, 3.7)	0.7630
HDL, umol/L	1.29 (1.06, 1.53)	1.40 (1.06, 1.67)	1.29 (1.16, 1.43)	1.29 (1.11, 1.56)	1.16 (0.98, 1.37)	0.0110
BMI, kg/m^2^	24.50 (22.60, 27.30)	22.70 (20.30, 24.50)	24.55(23.00 , 26.70)	25.70(23.90,27.40)	27.50 (24.60, 29.40)	< 0.0001
USAT,dB/cm/MHz	0.66 (0.54, 0.81)	0.52 (0.48, 0.57)	0.66 (0.61, 0.70)	0.75 (0.70, 0.80)	0.93 (0.86, 0.99)	< 0.0001
CAP,dB/m	253.60 ± 63.14	200.17 ± 45.93	252.07 ± 39.06	273.97 ± 26.42	324.80 ± 40.13	< 0.0001

numerical variables are presented as mean ± standard deviation or median and interquartile range, while categorical variables are represented as the number of patients and percentages. PLT, platelet; ALT, alanine aminotransferase; AST, aspartate transaminase; ALP, alkaline phosphatase; ALB, albumin; TP, total protein; DB, direct bilirubin; GLB, globulin; LDL, Low density lipoprotein; HDL, high-density lipoprotein; BMI, body mass index; USAT, ultrasound attenuation analysis; CAP, controlled attenuation parameter

### Correlation between USAT and CAP

**Fig. 2:**
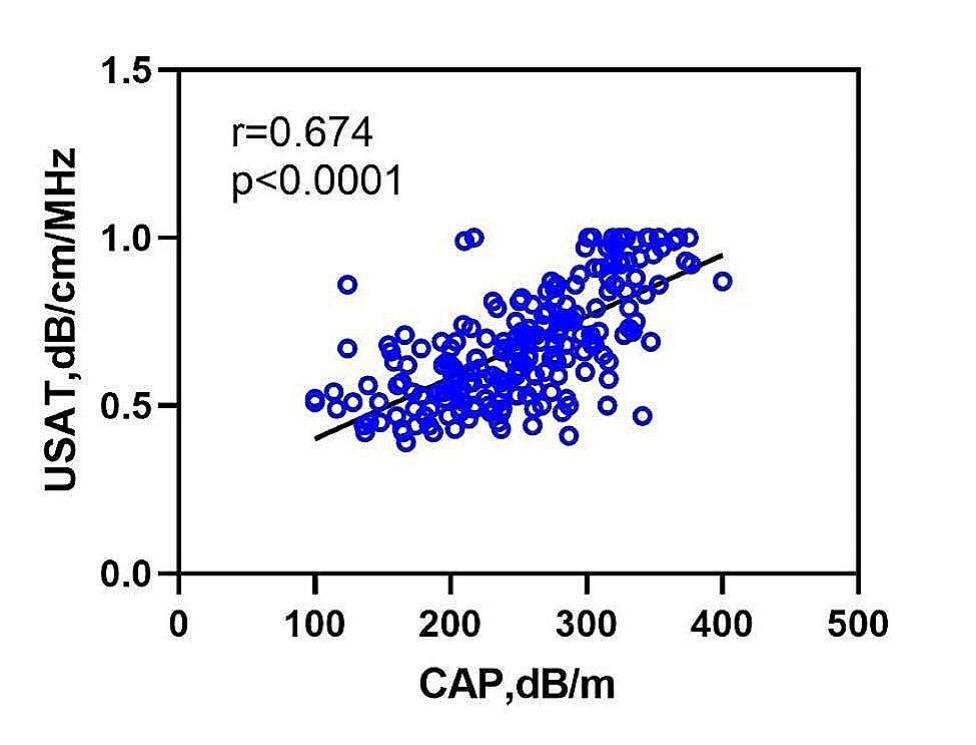
Pearson correlation between Ultrasonic Attenuation Analysis (USAT) and Controlled Attenuation Parameter (CAP). There is a linear correlation between USAT and CAP, demonstrating a strong correlation (correlation coefficient, *r* = 0.674, *p* < 0.0001). CAP, controlled attenuation parameter; USAT, ultrasound attenuation analysis

**Fig. 3:**
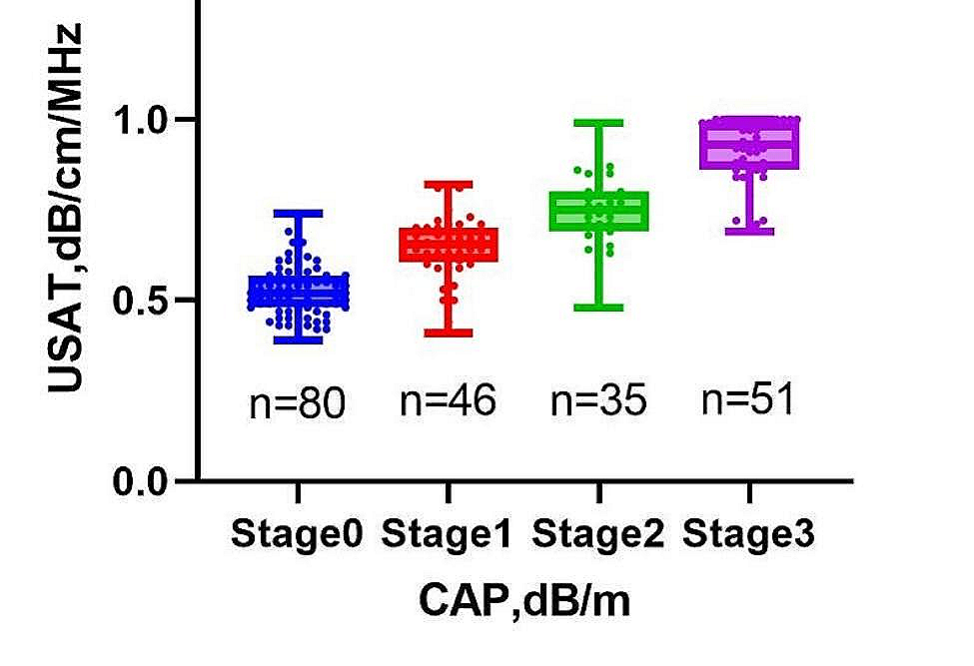
Scatter box plot illustrating the distribution of USAT values grouped by CAP in different stages of hepatic steatosis. (Kruskal-Wallis test shows that the p-values are all less than 0.05 among USAT values in hepatic steatosis stages 0, 1, 2, and 3). CAP, controlled attenuation parameter; USAT, ultrasound attenuation analysis

Measurements of USAT and CAP were valid for all 212 participants. There was a strong positive correlation between USAT and CAP (*r* = 0.674, *p* < 0.0001; Fig. 2). Figure 3 presents the distribution of USAT values at different stages of hepatic steatosis according to CAP. The average USAT values for Stage 0, Stage 1, Stage 2, and Stage 3 were 0.52, 0.66, 0.75, and 0.93 dB/cm/MHz, respectively. Kruskal-Wallis tests revealed significant differences in USAT values among Stage 0, Stage 1, Stage 2, and Stage 3 (*P* < 0.05).

### Comparison of diagnostic performance of USAT and CAP for hepatic steatosis grades

**Fig. 4:**
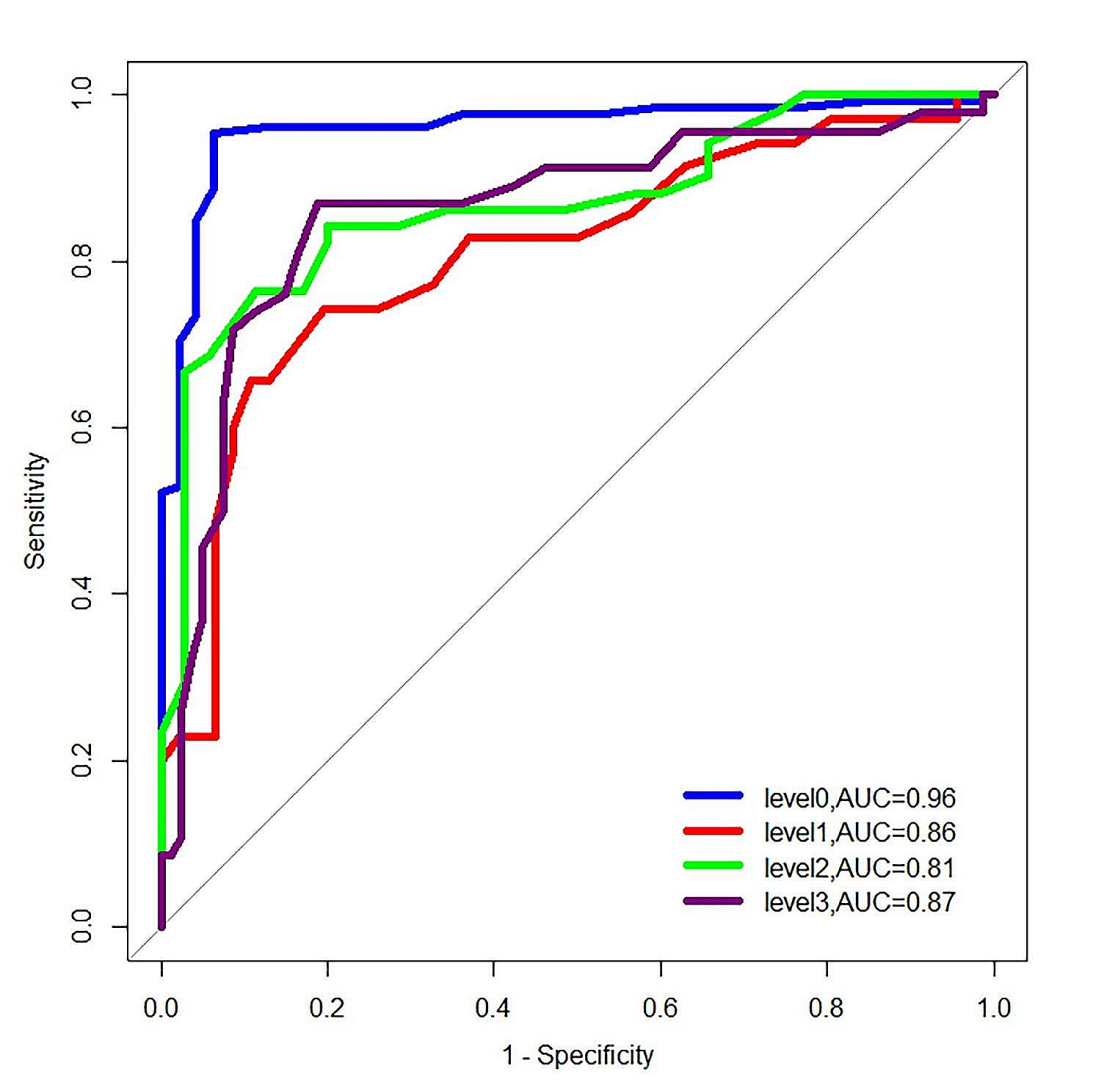
Displays the ROC curves for USAT in diagnosing hepatic steatosis at different stages. The AUC for USAT in excluding hepatic steatosis (level 0) and diagnosing mild (level 1), moderate (level 2), and severe (level 3) hepatic steatosis is 0.96, 0.86, 0.81, and 0.87, respectively. ROC, receiver operating characteristic; AUC, area under the curve; CAP, controlled attenuation parameter; USAT, ultrasound attenuation analysis

The diagnostic performance of USAT for detecting hepatic steatosis is as follows: the AUC (95% confidence interval, 95%CI) is 0.96 (0.93–0.99), the cut-off value (95%CI) is 0.54 (0.53–0.57) dB/cm/MHz, with a specificity of 95.5%, sensitivity of 93.6%, and accuracy of 95%. For the diagnosis of mild hepatic steatosis, the AUC (95%CI) was 0.86 (0.78–0.93), the cut-off value (95%CI) was 0.59 (0.58–0.63) dB/cm/MHz, with specificity, sensitivity, and accuracy of 87%, 81.3%, and 83.3%, respectively. In diagnosing moderate hepatic steatosis, the AUC (95%CI) was 0.81 (0.71–0.91), the cut-off value (95%CI) was 0.73 (0.68–0.74) dB/cm/MHz, with specificity, sensitivity, and accuracy of 65.7%, 89.1%, and 79.01%, respectively. Finally, for the diagnosis of severe hepatic steatosis, the AUC (95%CI) was 0.87 (0.79–0.95), the cut-off value (95%CI) was 0.86 (0.82–0.88) dB/cm/MHz, with specificity, sensitivity, and accuracy of 76.5%, 88.6%, and 81.4%, respectively (Table 2; Fig. 4).

**Table 2:** Diagnostic performance of graded hepatic steatosis

	AUC(95%CI)	Cut-off(95%CI)	Specificity (%)	Sensitivity (%)	Accuracy (%)	*P*
level 0	0.96(0.93–0.99)	0.54(0.53–0.57)	95.50	93.60	95.00	< 0.0001
level 1	0.86(0.78–0.93)	0.59(0.58–0.63)	87.00	81.30	83.30	< 0.0001
level 2	0.81(0.71–0.91)	0.73(0.68–0.74)	65.70	89.10	79.01	0.0311
level 3	0.87(0.79–0.95)	0.86(0.82–0.88)	76.50	88.60	81.40	0.0092

AUC, Area Under the Curve; CI, confidence interval; level 0 represents normal subjects vs. all hepatic steatosis patients. level 1 represents normal individuals vs. mild hepatic steatosis patients. level 2 represents patients with mild hepatic steatosis vs. patients with moderate hepatic steatosis. level 3 represents patients with moderate hepatic steatosis vs. patients with severe hepatic steatosis

## Discussion

Our study results indicate that the correlation between USAT and CAP, showing a strong correlation between them. The ALT, AST, ALP, HDL, BMI, USAT, and CAP are important variables related to hepatic steatosis, while age, gender, PLT, ALB, TP, DB, GLB, and LDL are not significant variables. More importantly, our study provides critical USAT cut-off values for clinical diagnosis and staging of hepatic steatosis. USAT, an innovative ultrasound technology, can be used for the diagnosis and quantitative grading of hepatic steatosis, potentially improving the accuracy and effectiveness of hepatic steatosis diagnosis and management in NAFLD patients.

Hepatic fat infiltration is a crucial histological feature of NAFLD and closely related to fibrosis and the progression of liver disease [20, 21]. Additionally, a recent study has underscored the intricate connection between NAFLD and Crohn’s disease (IBD). Notably, the prevalence of NAFLD among patients with IBD is substantial, reaching up to 23% [22]. Consequently, the early identification of steatosis and timely intervention are pivotal in averting the progression of the disease. A meta-analysis showed that ultrasound had a sensitivity of 84.8% and specificity of 93.6% for moderate to severe steatosis compared to histology [9]. Ultrasound, due to its low cost, safety, and convenience, has been considered the primary imaging technique for screening hepatic steatosis [4]. However, its limitations include operator dependence, subjectivity in assessment, and the inability to quantify fat infiltration, which restricts its clinical application [23]. CT is commonly used for the diagnosis and evaluation of hepatic steatosis, relying on the liver-to-spleen CT ratio and the relative density of hepatic vessels. Its sensitivity for diagnosing moderate to severe hepatic steatosis ranges from 46 to 72%, with specificity between 88% and 95% [24, 25]. However, there is overlap in CT values for different levels of hepatic fat infiltration, potentially reducing diagnostic accuracy [26]. Moreover, CT involves ionizing radiation, making it less suitable for widespread use in asymptomatic populations. MRI is highly accurate in diagnosing NAFLD, especially MRI-PDFF, which demonstrates excellent diagnostic performance for various levels of hepatic fat infiltration (AUROC 0.989, sensitivity 96%, specificity 100%) [27]. However, it is expensive and not widely accessible, limiting its use in screening.

CAP shows a strong correlation with the level of fat infiltration and effectively evaluates hepatic steatosis non-invasively [28]. Studies have demonstrated that CAP exhibits high diagnostic accuracy in the noninvasive evaluation of hepatic steatosis, achieving a predictive accuracy of over 80% for moderate and severe hepatic steatosis [29]. In a meta-analysis, the sensitivity for predicting mild hepatic steatosis was 68.8% with a specificity of 82.2%, for moderate hepatic steatosis it was 77.3% with a specificity of 81.2%, and for severe hepatic steatosis it was 88.2% with a specificity of 77.6%. This underscores CAP’s capability to identify mild, moderate, and severe hepatic steatosis [30]. However, the applicability of CAP is limited; for example, it is not suitable for patients with ascites, severe obesity, or acute liver injury [15, 31], and it can only assess liver stiffness and hepatic fat infiltration. In contrast, USAT can automatically avoid ductal structures, provide visual information using ultrasound imaging, and allow monitoring of hepatic fat content in the same patient at different times, displaying the patient’s prognosis with an intuitive “trend chart” tool. Therefore, USAT offers a new quantitative parameter for early assessment and staging of hepatic steatosis.

In our study, USAT demonstrated excellent diagnostic performance with AUCs above 0.80 for distinguishing different stages of hepatic steatosis. Notably, USAT had the highest AUC in discriminating the presence of hepatic steatosis (AUC: 0.96) and the second-highest AUC in detecting severe hepatic steatosis (AUC: 0.87), indicating that USAT excels in excluding hepatic steatosis and differentiating moderate and severe hepatic steatosis. The USAT cutoff values increased from stage 0 to stage 4, with high sensitivity and specificity. Therefore, USAT can differentiate various levels of hepatic steatosis. A recent study in NAFLD and CHB-infected patients assessed the accuracy of USAT in grading hepatic steatosis severity using CAP as a reference. This study found that in the entire population, the cut-off values for predicting mild, moderate, and severe hepatic steatosis with USAT were 0.62 dB/cm/MHz (AUC: 0.89), 0.66 dB/cm/MHz (AUC: 0.90), and 0.82 dB/cm/MHz (AUC: 0.90), and observed a high positive correlation between USAT and CAP values (*r* = 0.787, *P* < 0.001) [32]. Our study results are generally consistent with this study, except for a difference in moderate hepatic steatosis (in our study, AUC = 0.81 with a cutoff value of 0.73 dB/cm/MHz, while the study reported an AUC of 0.90 with a cutoff value of 0.66 dB/cm/MHz) [32]. This discrepancy might be related to the prevalence of moderate hepatic steatosis, which was 16.5% in our study cohort, compared to 36.5% in the study [32]. Furthermore, our study included NAFLD patients, whereas the study included patients with both NAFLD and CHB infection.

In addition, a meta-analysis involving 2735 patients determined the optimal cut-off values of CAP for predicting mild (S1), moderate (S2), and severe fatty liver (S3) to be 248 dB/m (AUC 0.823, sensitivity 68.8%, specificity 82.2%), 268 dB/m (AUC 0.865, sensitivity 77.3%, specificity 81.2%), and 280 dB/m (AUC 0.882, sensitivity 88.2%, specificity 77.6%) [30]. In our study, the CAP cutoff values for mild and moderate levels of hepatic steatohepatitic degeneration were slightly lower than those derived from the aforementioned meta-analysis, while those for severe levels were slightly higher. This difference may be associated with the distribution of BMI values in our study population, which mainly ranged from 22.7 to 27.5 kg/m², compared to the meta-analysis where BMI ranged from 23.6 to 27.6 kg/m². Furthermore, our study excluded diabetic subjects, whereas the meta-analysis included them. Previous studies have indicated that the critical values of CAP can be influenced by various factors, particularly in patients with non-alcoholic fatty liver disease (NAFLD)/NASH, diabetes, and those with a body mass index (BMI) ranging from 20 to 30 kg/m², per unit above or below 25 kg/m². It is further suggested to adjust these cutoff values by reducing the CAP value by 10 dB/m for NAFLD/NASH patients, by 10 dB/m for diabetic patients, and adjusting by deducting/adding 4.4 dB/m for each unit of BMI above/below 25 kg/m² within the range of 20–30 kg/m² [33, 34].

Our study has several limitations. First, it is a single-center study. Second, we did not use liver biopsy (the gold standard) as a reference to validate the accuracy of USAT in quantifying hepatic fat infiltration, which may partially affect the study results. However, as previously mentioned, we employed Controlled Attenuation Parameter (CAP) as a reference standard. CAP is a validated and recommended clinical method for quantifying hepatic steatosis, providing a relatively accurate measure of the severity of steatosis [29]. Third, the sample size in the study is relatively small, and based on this, we will conduct further research with a larger patient cohort.

## Conclusions

In summary, the attenuation coefficients determined through USAT show a strong correlation with CAP. In this study, we established USAT threshold values for various degrees of hepatic steatosis, using CAP as a reference, and validated the broad applicability of USAT in a cohort of patients potentially having steatosis. Due to its high diagnostic accuracy, USAT holds promise as a valuable tool for noninvasive assessment and grading of hepatic steatosis.

## Data Availability

No datasets were generated or analysed during the current study.
